# Long-Term Effects of Early Overnutrition in the Heart of Male Adult Rats: Role of the Renin-Angiotensin System

**DOI:** 10.1371/journal.pone.0065172

**Published:** 2013-06-03

**Authors:** Miriam Granado, Nuria Fernández, Luis Monge, Gonzalo Carreño-Tarragona, Juan Carlos Figueras, Sara Amor, Ángel Luis García-Villalón

**Affiliations:** 1 Department of Physiology, Faculty of Medicine, Universidad Autónoma de Madrid, Spain; 2 CIBER Fisiopatología de Obesidad y Nutrición, Instituto de Salud Carlos III, Madrid, Spain; Max-Delbrück Center for Molecular Medicine (MDC), Germany

## Abstract

To analyze the long-term effects of early overfeeding on the heart and coronary circulation, the effect of ischemia-reperfusion (I/R) and the role of the renin-angiotensin system (RAS) was studied in isolated hearts from control and overfed rats during lactation. On the day of birth litters were adjusted to twelve pups per mother (controls) or to three pups per mother (overfed). At 5 months of age, the rats from reduced litters showed higher body weight and body fat than the controls. The hearts from these rats were perfused in a Langendorff system and subjected to 30 min of ischemia followed by 15 min of reperfusion (I/R). The myocardial contractility (dP/dt) and the coronary vasoconstriction to angiotensin II were lower, and the expression of the apoptotic marker was higher, in the hearts from overfed rats compared to controls. I/R reduced the myocardial contractily, the coronary vasoconstriction to angiotensin II and the vasodilatation to bradykinin, and increased the expression of (pro)renin receptor and of apoptotic and antiapoptotic markers, in both experimental groups. I/R also increased the expression of angiotensinogen in control but not in overfed rats. In summary, the results of this study suggest that early overnutrition induces reduced activity of the RAS and impairment of myocardial and coronary function in adult life, due to increased apoptosis. Ischemia-reperfusion produced myocardial and coronary impairment and apoptosis, which may be related to activation of RAS in control but not in overfed rats, and there may be protective mechanisms in both experimental groups.

## Introduction

Obesity is a major health problem in the developed world [1]. Although obesity may affect several organs [2], mortality in this group is mainly associated with cardiovascular disease [3]. Obesity is a major risk factor for hypertension, heart failure and coronary heart disease [4], and weight reduction improves several of these cardiovascular risk factors [5].

There is evidence that cardiac pathology during obesity may be related to increased cardiomyocite apoptosis in this condition [6]. Apoptosis reduces the number of myocardial cells and increases the load of the remaining ones, leading to functional impairment of the heart. Among the pathways participating in increased cardiomyocite apoptosis in animal models of obesity, lipid accumulation [7], [8] and ceramide synthesis [9], or impairment of leptin signaling [10] have been proposed.

Another pathway possibly involved in the cardiac impairment associated with obesity may be the renin-angiotensin system [11]–[15]. In addition to circulating angiotensin II, this peptide is also produced in the cardiac tissue by a local renin-angiotensin system (RAS) [16]. Angiotensin II is reported to increase oxidative stress, leading to activation of inflammatory [17] and apoptotic [18] pathways. However, other components of the renin-angiotensin may have compensatory or protective effects. Recently, a cell receptor has been described which binds to renin and prorenin [19], and which activates antiapoptotic signaling pathways independently of angiotensin II production [20]. RAS may also be involved in myocardial damage during ischemia-reperfusion [21], which is more frequent is obese subjects [22]. As RAS may be altered in obesity, the pathophysiology of ischemia-reperfusion may be different in this condition, but the role of RAS in this context has been less studied than other mechanisms of ischemia-reperfusion injury.

Therefore, the aim of the present study is to analyze the role of the RAS in early overfeeding-induced alterations in heart function and coronary circulation. For that purpose we have used an experimental model of early overnutrition in rats since in humans, obesity often begins in childhood and adolescence, and continues during adult age [23], and the cardiac alterations caused by obesity may begin in childhood [24]. This experimental model is reported to induce an increase in food intake and weight gain during lactation that lasts after weaning [25], [26], and reproduces several characteristics the obesity originated in childhood in humans. As coronary ischemic disease is the main complication of obesity-related disorder in the heart, the role of the renin-angiotensin system was studied in control conditions and after ischemia-reperfusion in control and overfed rats.

## Materials and Methods

### Animals

#### Ethics statement

Sprague-Dawley rats were used for these studies (Harlan interfauna Ibérica S.A., Barcelona, Spain). All the experiments were conducted in accordance with the US National Institutes of Health Guide for the Care and Use of Laboratory Animals (NIH Publication No. 85–23, revised 1996) and in compliance with all relevant laws and regulations. The use of these animals was also approved by the Institute's Animal Care and Use Committee (Comité de Ética de la Investigación, Universidad Autónoma de Madrid).

After mating and pregnancy was confirmed, dams were housed individually and fed ad libitum until the end of pregnancy. On the day of birth 6 litters were adjusted to twelve pups per mother (controls, L12) and 6 litters were adjusted to three pups per mother (overfed, L3). After weaning on postnatal day 21, male rats were housed two per cage. Body weight and food intake were assessed weekly. On postnatal day 150 all rats were sacrificed by decapitation after being anesthetized with sodium pentobarbital (60 mg/kg). After decapitation blood and heart were collected. Visceral adipose tissue surrounding the epididymis and lumbar subcutaneous adipose tissue were dissected and immediately weighed after sacrifice.

### Heart Perfusion

The hearts were removed from the rats under anaesthesia with i.p. sodium pentobarbital (200 mg/kg) and following i.v. injection of heparin (1000 UI). Next, the ascending aorta was cannulated and the heart was subjected to retrograde perfusion with Krebs-Henseleit buffer (115 mM NaCl, 4.6 mM KCl, 1.2 mM KH_2_PO_4_, 1.2 mM MgSO_4_, 2.5 mM CaCl_2_, 25 mM NaHCO_3_ and 11 mM glucose) equilibrated with 95% oxygen and 5% carbon dioxide to a pH of 7.3–7.4. Perfusion was initiated in a non-recirculating Langendorff heart perfusion apparatus at a constant flow rate of 11–15 ml/min to provide a basal perfusion pressure of approximately 70 mmHg. Both the perfusion solution and the heart were maintained at 37°C. Coronary perfusion pressure was measured through a lateral connection in the perfusion cannula and left ventricular pressure was measured using a latex balloon inflated to a diastolic pressure of 5–10 mmHg, both connected to Statham transducers (Statham Instruments, Los Angeles, California). Left ventricular developed pressure (systolic left ventricular pressure minus diastolic left ventricular pressure), the first derivate of the left ventricular pressure curve (dP/dt) and heart rate were calculated from the left ventricular pressure curve. These parameters were recorded on a computer using Chart 5 v5.4.1 software and the PowerLab/8SP data acquisition system (ADInstruments, Colorado Springs, Colorado).

After a 15 min equilibration period with constant flow perfusion, the hearts were exposed to global zero-flow ischemia for 30 min and reperfused for 15 min at the same flow rate used before ischemia. The duration of ischemia and reperfusion were chosen on the basis of previous studies demonstrating decreases in the endothelium-dependent coronary relaxation without alteration of endothelium-independent coronary relaxation [27], [28]. The control hearts were perfused during a similar total time (60 min) at constant flow without ischemia. After I/R or perfusion during 60 min the coronary vasoconstriction to angiotensin II or the vasodilatation to bradykinin was recorded. Angiotensin II was injected into the perfusion cannula with an infusion pump over 3 min at a constant rate to reach a final concentration of 10^−12^–10^−7^ M. The relaxation to bradykinin was recorded after precontracting the coronary arteries with the thromboxane A_2_ analogue U46619. First, 10^−8^ M U46619 was added to the perfusion solution and the concentration was increased progressively until a contractile tone of ∼120–140 mmHg was obtained. The concentrations of U46619 required to achieve this effect were 1×10^−8^ to 3×10^−8^ M in control conditions and 5×10^−8^ to 2×10^−7^ M after ischemia-reperfusion. When the contractile tone reached a stable level, bradykinin was injected into the perfusion cannula over 2 min at a constant rate to reach a final concentration of 10^−9^–10^−6^ M). As the experiments were performed at a constant flow rate, the coronary perfusion pressure provides a measure of the perfusion resistance and characterizes the contraction or relaxation of the coronary arteries.

### Tissue Homogenization and Protein Quantification

Heart tissue was homogenized in 500µl of radioimmunoprecipitation assay lysis buffer with an EDTA-free protease inhibitor cocktail (Roche Diagnostics, Mannheim, Germany). After homogenization, samples were centrifuged at 14,000 rpm for 20 min at 4°C. Supernatants were transferred to a new tube and protein concentration was estimated by Bradford protein assay.

### Immunoblotting

In each assay the same amount of protein was loaded in all wells (75 µg) and resolving gels with different amount of SDS-acrylamide gels (8–12%) were used depending on the molecular weight of the protein. After electrophoresis proteins were transferred to polyvinylidine difluoride (PVDF) membranes (Bio-Rad) and transfer efficiency was determined by Ponceau red dyeing. Filters were then blocked with Tris-buffered saline (TBS) containing 5% (w/v) non-fat dried milk and incubated with the appropriate primary antibody; caspase-3 (Cell Signalling), caspase-6 (Medical Biological Laboratories), caspase-8 (Neomarkers), Bcl-2 (Thermo Scientific), Hsp-70(Stressgen Bioreagents), iNOS (BD Biosciences), COX-2 (Cell Signalling). Membranes were subsequently washed and incubated with the corresponding secondary antibody conjugated with peroxidase (1∶2000; Pierce, Rockford, IL, USA). Bound peroxidase activity was visualized by chemiluminescence and quantified by densitometry using BioRad Molecular Imager ChemiDoc XRS System. All blots were rehybridized with β-tubuline (Sigma-Aldrich) to normalize each sample for gel-loading variability. All data are normalized to control values on each gel.

### RNA Preparation and Purification and Quantitative Real-time PCR

Total RNA was extracted from the myocardium according to the Tri-Reagent protocol [29]. cDNA was then synthesized from 1 µg of total RNA using a high capacity cDNA reverse transcription kit (Applied Biosystems, Foster City, CA, USA).

### Quantitative Real-time PCR

Angiotensinogen, angiotensin II receptor 1a (AgtR1a), angiotensin II receptor 2 (AgtR2) and pro-renin receptor (RR) mRNAs were assessed in heart samples by quantitative real-time PCR. Quantitative real-time PCR was performed by using assay-on-demand kits (Applied Biosystems) for each gene: Angiotensinogen (Rn00593114 m1), AgtR1a (Rn02758772s1), AgtR2 (Rn00560677s1) and RR (Rn01430718 m1). TaqMan Universal PCR Master Mix (Applied Biosystems) was used for amplification according to the manufacturer’s protocol in a Step One machine (Applied Biosystems). Values were normalized to the housekeeping gene 18S (Rn01428915). According to manufacturer’s guidelines, the ΔΔCT method was used to determine relative expression levels. Statistics were performed using ΔΔCT values [30].

### Statistical Analysis

Values are expressed as the mean (± SEM). Body and organ weight and angiontensin and leptin serum levels were compared in rats from control or reduced litters by unpaired Student’s t test. Concentration-response curves to angiotensin II and bradykinin in rats from control or reduced litters, after ischemia-reperfusion or control perfusion, were compared by three-way ANOVA, and the pD2 of the curves (negative logarithm of the EC50, calculated from each curve by geometric interpolation) were compared by two-way ANOVA. Hemodynamic data from the hearts, and western blot and PCR data in rats from control or reduced litters, after ischemia-reperfusion or control perfusion, were also compared by two-way ANOVA. Each ANOVA was followed by post-hoc Bonferroni test. A p value of <0.05 was considered significant.

### Drugs and Chemicals

The following substances were all obtained from Sigma (Tres Cantos, Madrid, Spain): Angiotensin II acetate; bradykinin acetate and 9,11-dideoxy-1a,9a-epoxymethanoprostaglandin F_2α_ (U46619).

## Results

### Growth Curve and Food Intake

**Figure 1:**
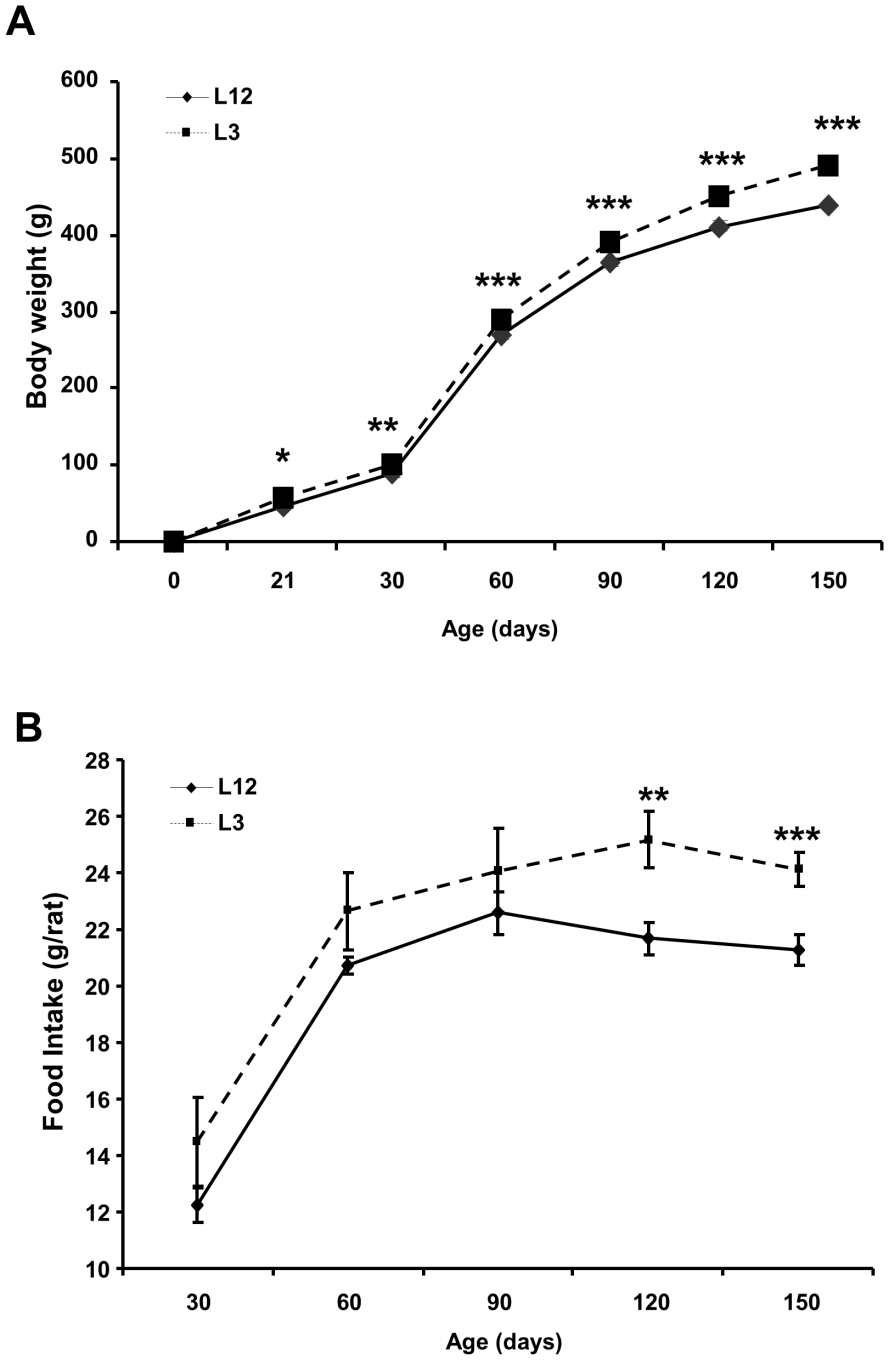
Body weight (g) and food intake from control (L12) and overfed (L3) rats. Data are represented as mean ±S.E.M (n = 18–20), and the data was compared by unpaired Student’s t test. *P<0.05 **P<0.01 **P<0.001 vs L12.

Rats raised in small litters had increased body weight compared with rats raised in control litters at one (P<0.05), two (P<0.01), three (P<0.001), four (P<0.001) and five (P<0.001) months of age (Figure 1A). Food intake was unchanged between control and overfed rats at one, two and three months of age (Figure 1B) but it was increased in L3 rats compared to L12 rats at the ages of four and five months (P<0.05 and P<0.01 respectively).

### Fat, Skeletal Muscle, Heart, Kidney and Adrenal Glands Weights

L3 rats had increased epidydimal and subcutaneous fat weights (P<0.001 for both, Table 1). Muscle mass (gastrocnemius and soleus weights) was also increased in rats raised in small litters compared to rats raised in control litters (P<0.01, Table 1). Adrenal glands weight was unchanged between both experimental groups but kidney and heart weights were significantly higher in L3 rats compared to L2 (P<0.05).

**Table 1:** Epidydimal Fat, subcutaneous fat, gastrocnemius, heart, kidneys and adrenal weights from control (L12) and overfed (L3) rats. Data are represented as mean **±** SEM.

	L12	L3
**Epidydimal Fat (mg)**	4717±181	6635±298 ***
**Subcutaneous Fat (mg)**	2771±152	3621±169 ***
**Gastrocnemius (mg)**	2438±37	2560±40*
**Soleus (mg)**	186±4	195±5*
**Heart (mg)**	2235±43	2434±60*
**Kidneys (mg)**	1356±22	1554±30 *
**Adrenal glands (mg)**	50±1.06	50±1.5

n = 18–20. Data were compared by unpaired Student’s t test.

* P<0.05 vs L12;

*** P<0.001 vs L12.

### Leptin and Angiotensin II Serum Levels

**Figure 2:**
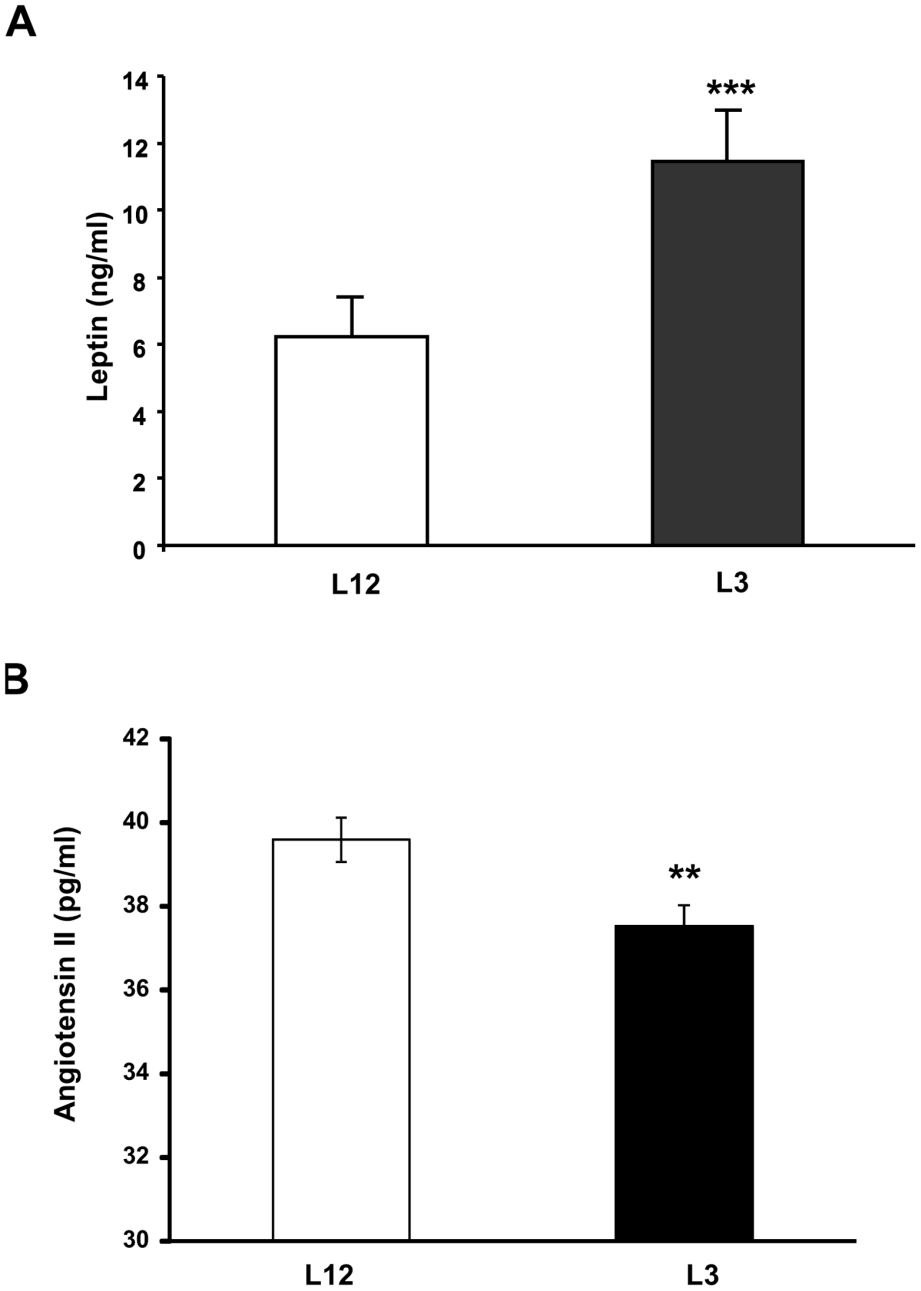
Leptin and angiotensin II plasma levels in L12 (Controls) and L3 (Overfed) rats. Data are represented as mean ±S.E.M (n = 10), and the data was compared by unpaired Student’s t test. *P<0.05 **P<0.01 vs L12.

Leptin serum levels were increased in early overfed rats compared to controls (P<0.001, Figure 2A). On the contrary angiotensin II serum levels were decreased in L3 rats compared to L12 rats (P<0.01, Figure 2B).

### Hemodynamic Parameters in the Perfused Hearts

Before ischemia-reperfusion coronary in the perfused rats, coronary perfusion pressure, left developed intraventricular pressure and heart rate were similar in the rats from L12 or L3 litters, but dP/dt was significantly lower (P<0.05) in the hearts of the L3 rats. Ischemia-reperfusion induced a significant decrease (P<0.01) in left ventricular developed pressure, maximal dP/dt and heart rate in hearts from both L12 and L3 rats (Table 2).

**Table 2:** Hemodynamic values in perfused hearts from control (L12) or overfed (L3) rats before and after 30 min of ischemia and 15 min of reperfusion (I/R).

	Coronary perfusionpressure (mmHg)	Left intraventricular developedpressure (mmHg)	dP/dt (mmHg/s)	Heart rate (beats/min)
L12 control (n = 14)	74±2	136±7	3421±218	252±6
L12+ I/R (n = 13)	74±2	67±13*	1431±245*	208±14*
L3 control (n = 13)	75±1	110±7	2614±209#	246±11
L3+ I/R (n = 12)	78±3	47±7*	772±113*	193±13*

Data are represented as means ±SEM. n = number of hearts.

The data was compared by two-way ANOVA and Bonferroni's test.

* (P<0.01) I/R vs. control;

# (P<0.05) L12 vs. L3.

### Coronary Vasoconstriction to Angiotensin II

**Figure 3:**
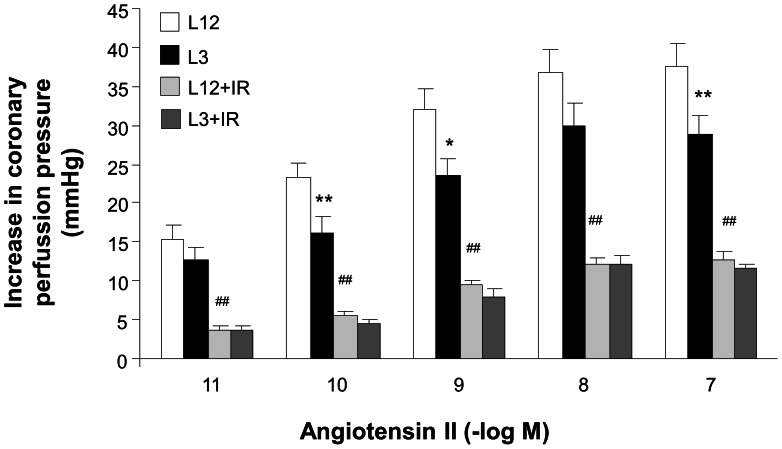
Coronary vasoconstriction to angiotensin II (10^−11^–10^−7^ M) in perfused hearts from control (L12) or overfed (L3) rats, with or without 30 min of ischemia and 15 min of reperfusion (I/R). *P<0.05 **P<0.01 L12 vs.L3 rats. ^#^P<0.01 I/R vs. control. Values are represented as mean ±S.E.M., and the data was compared by three-way ANOVA and Bonferroni's test. n number of hearts.

Injection of angiotensin II into the coronary circulation induced concentration-dependent increases of the coronary perfusion pressure in the perfused hearts from both L12 and L3 rats, but these increases were smaller in the L3 rats compared with the controls (P<0.001 by three-way ANOVA), although pD2 values were not different (10.41±0.16 vs. 10.63±0.20, respectively). After ischemia-reperfusion, the vasoconstriction to angiotensin II was reduced (P<0.001 by three-way ANOVA) similarly in both experimental groups (pD2 9.53±0.18 and 9.81±0.21, respectively, p<0.01 by two-way ANOVA) (Figure 3).

### Coronary Vasodilatation to Bradykinin

**Figure 4:**
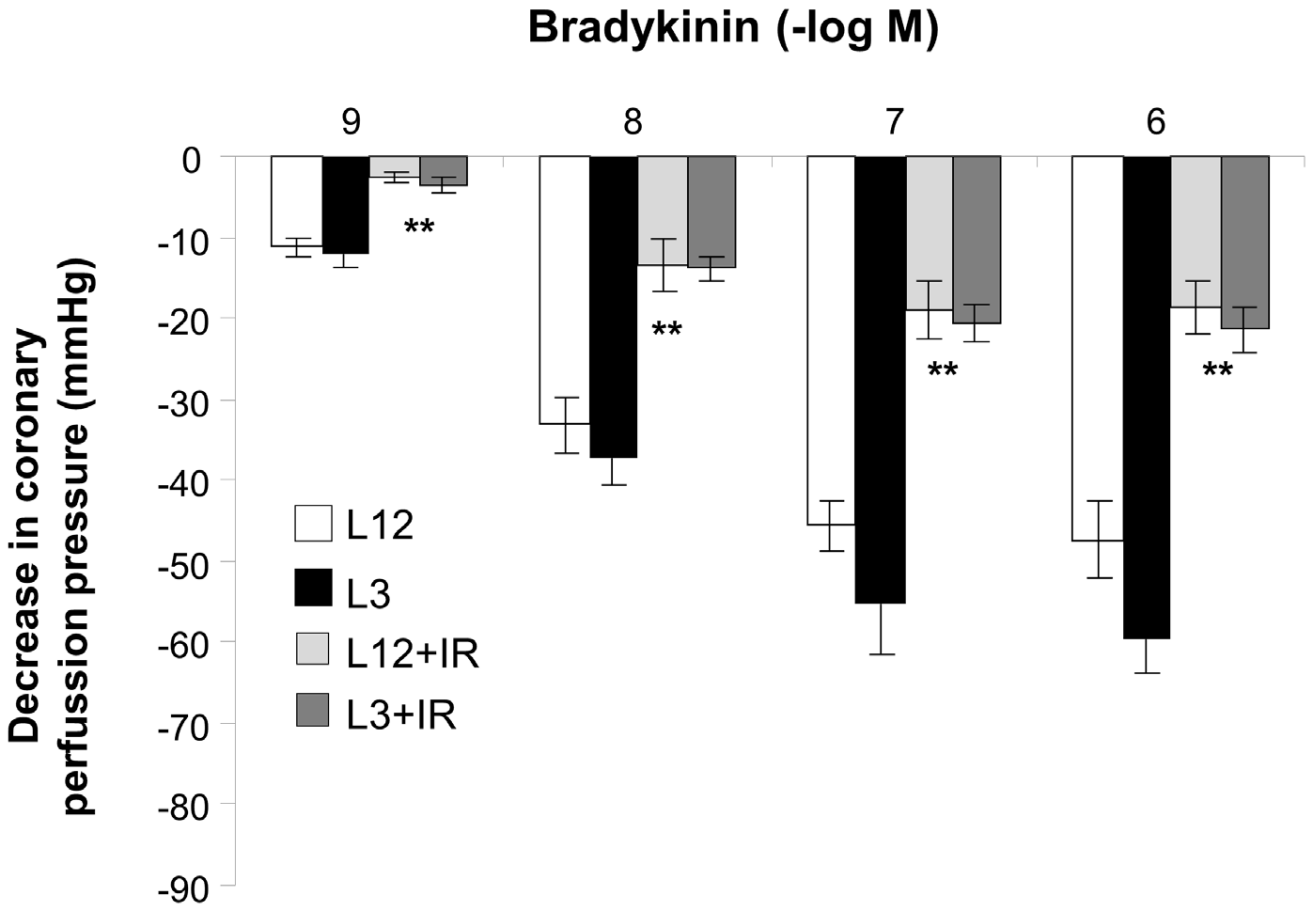
Coronary vasodilatation to bradykinin (10^−9^–10^−6^ M) after precontraction with U46619 in perfused hearts from control (L12) or overfed (L3) rats, with or without 30 min of ischemia and 15 min of reperfusion (IR). *P<0.01 I/R vs. control. Values are represented as mean ±S.E.M, and the data was compared by three-way ANOVA and Bonferroni's test. n number of hearts.

After precontraction of the coronary circulation with U46619, injection of bradykinin induced a significant reduction in the coronary perfusion pressure. This effect of bradykinin was similar in the hearts from L12 (pD2 8.52±0.18) and L13 (pD2 8.48±0.17) litters, and was similarly reduced (P<0.001 by three-way ANOVA) after ischemia-reperfusion in both experimental groups (PD2 8.61±0.06 and 8.67±0.07, respectively, P<0.01 by two-way ANOVA) (Figure 4).

### Angiotensinogen, AgtR1a, AgtR2, Renin and RR Gene Expression

**Figure 5:**
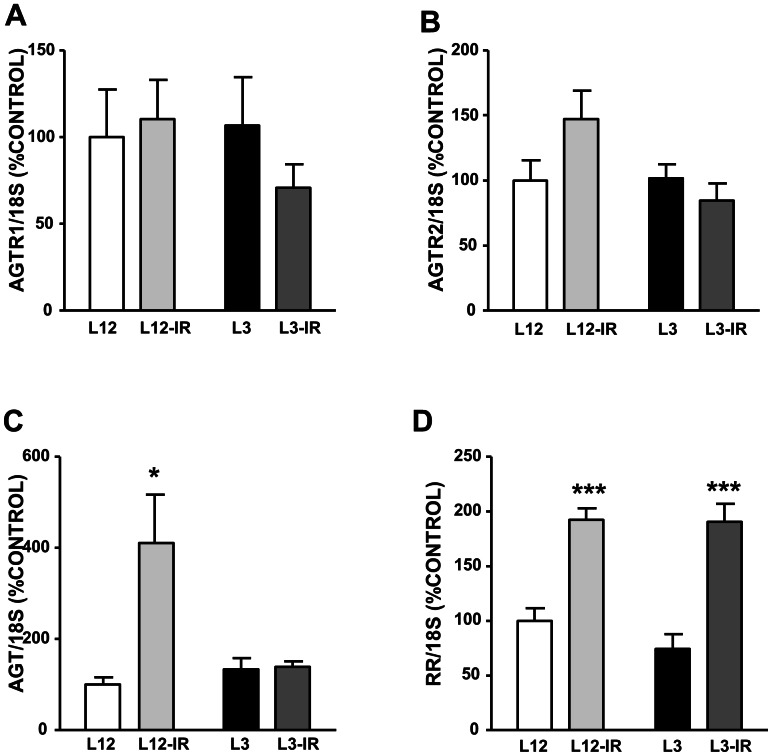
Gene expression of angiotensinogen (A), angiotensin receptor type 1a (AgtR1a, (B)), angiotensin receptor type 2 (AgtR2, (C)) and pro-renin receptor (RR, (D)) in the myocardium of control (L12) or overfed (L3) rats subjected or not to 30 min of ischemia and 15 min of reperfusion (IR). Values are represented as mean ±S.E.M (n = 6/group), and the data was compared by two-way ANOVA.*P<0.05 vs L12; ^#^P<0.05 vs L12-IR.

Litter reduction did not modify the gene expression of AgtR1, AgtR2, angiotensinogen and pro-renin receptor (Figures 5A, 5B, 5C and 5D respectively). However ischemia-reperfusion increased angiotensinogen mRNA expression in L12 rats (P<0.05, Figure 3C) and pro-renin receptor mRNA both in L12 and in L3 rats (P<0.001, Figure 5D). Renin gene expression was not detected neither in the myocardium of rats raised in control litters nor in the myocardium of rats raised in small litters.

### Bradikinin Receptor B1 (BDKRB1) and Bradikinin Receptor B2 Gene Expression (BDKRB2)

The gene expression of BDKRB1 was unchanged in response to both ischemia-reperfusion and litter reduction. (L12 = 100±14; L12+I/R = 108±12; L3 = 106±16; L3+I/R = 83±9). Likewise there were no changes in the gene expression of BDKRB2 between the experimental groups (L12 = 100±15; L2+I/R = 130±18; L3 = 97±15; L3+I/R = 90±25).

### Apoptotic Markers in the Myocardium

**Figure 6:**
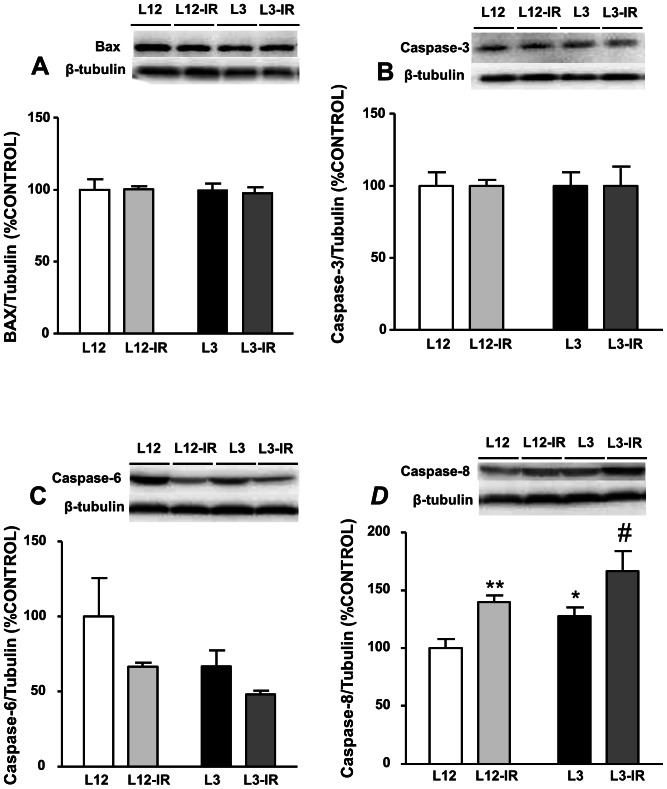
Levels of Bcl-2–associated×protein (Bax, (A)), caspase-3 (B), caspase-6 (C) and caspase-8 (D) in the myocardium of control (L12) or overfed (L3) rats subjected or not to 30 min of ischemia and 15 min of reperfusion (IR). Values are represented as mean ±S.E.M (n = 4–6/group), and the data was compared by two-way ANOVA. *P<0.05 vs L12; **P<0.01 vs L12; ^#^P<0.05 vs L12-IR; ^###^P<0.001 vs L12-IR.

Neither litter reduction nor ischemia-reperfusion induced a significant effect in Bcl-2–associated×protein (Bax), caspase-3 or caspase-6 levels in the myocardium (Figures 6A, 6B and 6C respectively). However, the content of the activated caspase-8 in the myocardium was significantly increased in response to both litter reduction (P<0.05) and I/R (P<0.001) (Figure 6D).

### Anti-apoptotic Markers in the Myocardium

**Figure 7:**
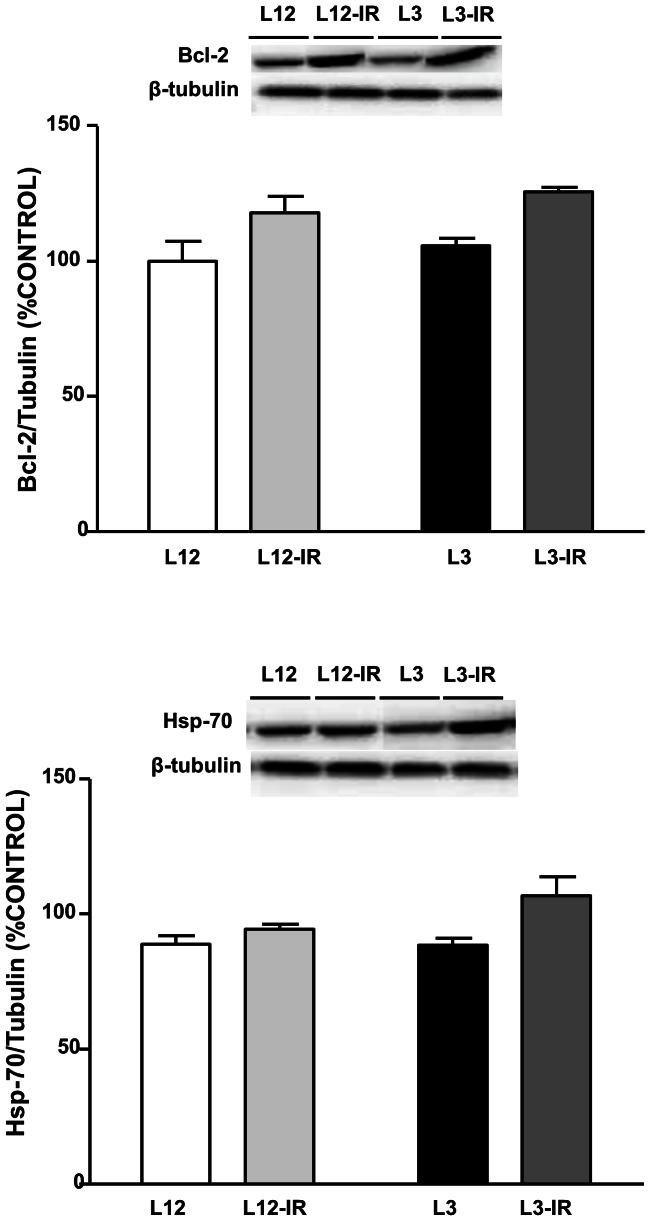
Levels of B-cell lymphoma 2 (Bcl-2, (A)) and heat shock protein 70 (Hsp-70, B)) in the myocardium of control (L12) or overfed (L3) rats subjected or not to 30 min of ischemia and 15 min of reperfusion (IR). Values are represented as mean ±S.E.M (n = 4–6/group), and the data was compared by two-way ANOVA. ^+^P<0.05 vs L3.

The levels of the anti-apoptotic markers B-cell lymphoma 2 (Bcl-2) and heat shock protein 70 (Hsp-70) were unchanged in response to litter reduction (Figures 7A and 7B). However there was an effect of I/R increasing the myocardial content of these two anti-apoptotic proteins (P<0.05).

### Inflammatory Markers in the Myocardium

The content of the inflammatory markers iNOS and COX-2 in the heart was unchanged in response to both early overfeeding and I/R (data not shown).

## Discussion

The present study suggests that early overnutrition during lactation induces changes in the adult heart. The rats from reduced litters had greater food intake and weight gain than control rats, and these changes persisted after weaning, so at the age of 5 months the rats from reduced litters still had higher body weight than controls. This was correlated with greater proportion of visceral and subcutaneous fat, higher plasma leptin levels and lower skeletal muscle mass. Overfeeding during early life may induce permanent changes, such as impaired response of neurons in arcuate and ventromedial hypothalamic nuclei to the anorexigenic effects of leptin and insulin [25], [26], resulting in altered metabolic conditions in adult life. In humans, obesity in childhood usually persists in adult life, with the corresponding metabolic alterations. Increased waist circumference and body mass index during adolescence are correlated positively with cardiovascular risk factors such as high blood pressure and low high-density lipoprotein cholesterol during young adulthood [31]. The results of the present study agree with the hypothesis that the alterations induced by early overfeeding result in cardiovascular impairment later in life. The hearts from overweight rats presented a reduced myocardial contractility compared with control rats, as indicated by a lower dP/dt. This impaired myocardial contractility may be associated with a reduced number of myocardial cells due to increased apoptosis, as we have found increased expression of the apoptosis marker caspase 8 in the myocardial tissue from overweight rats. Likewise, apoptosis has been shown to be increased in the myocardium of pigs with metabolic syndrome [32] and Zucker obese rats [33], [34]. We had previously described that different apoptotic markers such as caspase-6 and caspase-8, as well as different markers involved in survival and proliferation such as Bcl-2 and Hsp-70 were up-regulated in response to litter reduction at the age of 21 days [35]. In addition, most of these changes were exacerbated in hearts from overfed rats exposed to ischemia-reperfusion. Likewise it has been recently reported that early structural changes in the heart induced by perinatal malnutrition are no longer evident at the age of 6 months [36]. The reason why these markers are no longer modified in adulthood deserves further investigation. However, it is possible that alterations in cardiomyocyte population in response to early overnutrition are more evident at early ages as these cells are immature and are still differentiating during the postnatal period [37].

Moreover, this myocardial dysfunction may be associated with impairment of myocardial and coronary regulation pathways. One of these pathways involved may be the RAS, which plays an important role in the heart and coronary circulation [16]. We have found in the present study that the coronary vasoconstriction to angiotensin II was reduced in the hearts of overfed rats, and this was associated with lower plasma levels of angiotensin II in these rats. Obesity induced by altered diet may be associated with increased plasma angiotensin II [38], [39], this discrepancy may be due to the different model of obesity used (litter restriction vs. altered diet). Although the angiotensin receptors AgtR1 and AgtR2 were not modified, the reduced coronary response to angiotensin II suggest impaired post-receptorial mechanisms. Previous observations in our laboratory have shown that at weaning (21 days), rats with overfeeding induced by the same procedure as in the previous study presented augmented expression of both AgtR1 and AgtR2 [35]. This was not observed in the rats aged 5 months, which might suggest that during maturation a reduction of the expression of these receptors compared with the young age takes place, and therefore a reduced activity and/or responsiveness of the RAS in adult overfed rats. A possible explanation for these results is that although activation of the RAS has been described in early obesity [40], this may lead to exhaustion and impairment of this system later in life.

On the contrary, the coronary endothelium-dependent vasodilatation to bradykinin was not modified by overfeeding. This contrasts with studies in mesenteric [41], skeletal muscle [42], kidney [43] or cerebral [44] circulations, in which obesity reduces endothelium-dependent relaxation. In the coronary circulation, the effect of obesity on the endothelium-dependent relaxation varies, with reduction [45]–[47], no change [48], [49] or even increase [50] of the relaxation to acetylcholine or bradykinin. It has been proposed that the coronary circulation could be partly resistant to endothelial dysfunction during obesity [51]. Increased myocardial work due to higher body mass in obese subjects [52] should produce increased coronary blood flow and shear stress on the endothelial surface, which stimulates release of endothelial vasodilator factors such as nitric oxide [53]. Progression of metabolic alterations may produce endothelial lesion later in life [47], [54] and coronary endothelial dysfunction may appear in the present model of overfeeding at a more advanced age.

As myocardial isquemia is more frequent in obese subjects [22], we have studied also the effects of ischemia-reperfusion in the hearts of overfed and control rats. In controls, ischemia-reperfusion induced a reduction of myocardial contractility, coronary vasoconstriction to angiotensin II and coronary endothelium-dependent vasodilatation to bradykinin. This agrees with previous studies [27], [28], [55], suggesting that this condition may cause injury of myocardial, coronary smooth muscle and endothelial cells, respectively, and this injury was correlated with increased expression of apoptotic markers after ischemia-reperfusion. Although the degree of impairment induced by ischemia-reperfusion on myocardial and coronary function was similar in overfed and control rats, the mechanisms of this impairment may differ. In control rats, ischemia-reperfusion induced a marked increase in the expression of angiotensinogen. There is evidence of the presence of a cardiac RAS [16], which may produce locally angiotensin II from angiotensinogen in the heart. Renin activity may be present in myocardial tissue of perfused rat hearts [56], [57], which probably comes from plasma renin [58], but may remain in the tissue for a long time after it is removed from the perfusate [59], and may produce angiotensin I and II from angiotensinogen synthetized locally [60]. Therefore our results suggest that there may be increased production of angiotensin II during ischemia-reperfusion in control rats, as it has been observed previously [38]. Studies with longer reperfusion times [61]–[63] may also show increase in angiotensin receptors. As angiotensin II mediates oxidative stress [17], which can lead to apoptosis [18], this increased production may partly be responsible of the myocardial and coronary injury during ischemia-reperfusion. In support of this, antagonist of AT1 receptors have a protective effect during myocardial ischemia-reperfusion [64]–[66]. However, in the hearts of overfed rats ischemia-reperfusion did not increase angiotensinogen expression, which suggests that in these hearts the impairment by ischemia-reperfusion is not related to angiotensin II production but due to other mechanisms. A previous study [38] has found that ischemia-reperfusion increased myocardial angiontensin II also in diet-induced obese rats, less than in control rats. This agrees with the lower activation of the RAS in the adult overfed rats hypothesized in this study.

Ischemia-reperfusion also increased expression of the (pro)renin receptor in the hearts of both overfed and control rats. This receptor has been recently described [67] and by binding to it renin and (pro)renin may have effects independent of angiotensin II production, by receptor-mediated activation of intracellular pathways [68]. Expression of (pro)renin receptor in the heart is increased in pathological conditions such as congestive heart failure [69] or in stroke-prone hypertensive rats [70]. This receptor activates expression of antiapoptotic mechanisms [20], and indeed we have observed an increase of antiapoptotic markers expression during ischemia in the hearts of overfed and control rats. Thus, it may be hypothesized that the (pro)renin receptor may have a protective effect during ischemia-reperfusion, by partly reducing the apoptosis-mediated tissue injury in this condition.

In summary, the results of this study suggest that early overnutrition induces reduced activity of the RAS and impairment of myocardial and coronary function in adult life, due to increased apoptosis. Overfeeding may also affect the mechanisms of myocardial injury during ischemia-reperfusion.
